# Non-EU migrant doctors in Ireland: applying a typology of health worker migration

**DOI:** 10.1186/1472-6963-14-S2-O21

**Published:** 2014-07-07

**Authors:** Niamh Humphries, Sara McAleese, Ella Tyrrell, Steve Thomas, Charles Normand, Ruairi Brugha

**Affiliations:** 1 Department of Epidemiology, RCSI, Dublin, Ireland; 2 Centre for Health Policy and Management, Trinity College Dublin, Ireland

## Background

Research on health worker migration in the Irish context has previously sought to categorize migrant health workers by country or region of training (e.g. non-EU nurses or doctors), by migration channel or mechanism (e.g. actively recruited nurses). This paper applies a recently developed typology of health worker migration [1], to the experiences of non-EU migrant doctors in Ireland and considers the value of a typology of health worker migration to human resource management internationally.

## Materials and methods

The paper draws on quantitative (N=37) and qualitative (N=337) data collected from non-EU migrant doctors in Ireland between 2011 and 2013.

## Results

While non-EU migrant doctors can be categorized according to the typology [1], in the Irish context, a cross-cutting theme of frustration emerged from doctors in almost all categories. In addition to ‘career oriented migrants’ expressing frustration at poor career progression, were ‘returners’ who could not return home because of their limited career progression in Ireland and ‘livelihood migrants’ frustrated with weak career progression.

## Conclusions

Employing a typology of health worker migration can facilitate a more comprehensive understanding of the migrant medical workforce, a necessary prerequisite for the development of useful policy tools [2]. However, our findings suggest that migrant health workers cannot be divorced from the health system context in which they work in the destination country, and perhaps this should be incorporated into the typology.
